# Diffusion-weighted imaging as a potential non-gadolinium alternative for immediate assessing the hyperacute outcome of MRgFUS ablation for uterine fibroids

**DOI:** 10.1038/s41598-024-60693-4

**Published:** 2024-04-29

**Authors:** Yaoqu Huang, Shouguo Zhou, Yinghua Su, Zhuochao Pang, Shihua Cai

**Affiliations:** 1 Center of MRgFUS, Foshan Hospital of Traditional Chinese Medicine, Guangzhou University of Chinese Medicine, No.6 Qinren Road, Foshan, 528000 Guangdong China; 2 Chancheng High-Tech District Hospital of Foshan Hospital of Traditional Chinese Medicine, Foshan, 528000 Guangdong China; 3 Department of Gynecology, Foshan Hospital of Traditional Chinese Medicine, Guangzhou University of Chinese Medicine, Foshan, 528000 Guangdong China

**Keywords:** Urogenital diseases, Magnetic resonance imaging

## Abstract

The aim of this study was to investigate the value of diffusion-weighted imaging (DWI) as a potential non-gadolinium alternative for promptly assessing the hyperacute outcome of magnetic resonance-guided focused ultrasound (MRgFUS) treatment for uterine fibroids. In this retrospective study we included 65 uterine fibroids from 44 women, who underwent axial DWI (b-value: 800 s/mm^2^) and contrast-enhanced (CE) MR within 15 min post-ablation. Two blinded observers independently reviewed the DWI findings of ablated necrotic lesions and measured their volumes on DWI and CE images. The post-ablation DWI images revealed clear depiction of ablative necrotic lesions in all fibroids, which were classified into two types: the bull’s eye sign (type 1) and the bright patch sign (type 2). The inter-observer intraclass correlation coefficient for classifying DWI signal types was 0.804 (*p* < 0.001). Volumetric analysis of ablated necrosis using DWI and CE T1-weighted imaging showed no significant variance, nor did the non-perfused volume ratios (all *p* > 0.05). Bland–Altman analysis revealed a mean difference of 2.38% and 1.71% in non-perfused volume ratios between DWI and CE, with 95% limits of agreement from − 19.06 to 23.82% and − 18.40 to 21.82%, respectively. The findings of this study support the potential of DWI as a viable non-gadolinium alternative for evaluating the hyperacute outcomes of MRgFUS ablation in uterine fibroids.

## Introduction

Magnetic resonance-guided focused ultrasound (MRgFUS), also known as magnetic resonance-guided high-intensity focused ultrasound, is is a burgeoning, effective, and non-invasive technique for thermally ablating uterine fibroids^1–3^. Prompt evaluation of the ablation rate during MRgFUS is vital; it facilitates early identification of under-ablated areas and informs decisions for supplementary treatments to optimize outcomes. A higher ablation ratio—calculated as the ratio of abated volume to total fibroid volume—is linked to enduring clinical benefits^4,5^. The most common approach for ablated tissue detection post-ablation employs T1-weighted MR imaging with gadolinium-based contrast agents^6,7^. On gadolinium contrast-enhanced (CE) T1-weighted imaging, the region of coagulative necrosis created by ablation is considered non-enhancing and is referred to as the non-perfused volume (NPV). Although CE T1-weighted is reliable, it is not recommended for repeated use at short intervals due to some concerns associated with gadolinium-based contrast agents. A second session may be performed for inadequate treatment but not until the gadolinium-based contrast agent has effectively been eliminated, as the FDA has cautioned that the high-energy beam may release toxic free gadolinium^2^. The contrast agent dose constraints and risk of allergic reactions or gadolinium deposition also limit the repeated use of it^8^. In addition, the use of a paramagnetic MR contrast agent prior to MR-HIFU treatment may influence the accuracy of the PRFS MR thermometry^9^. Thus, CE T1-weighted imaging could not help physicians to decide whether to end or continue the MRgFUS procedure because it is recommended only at the end of treatment, and a method for repeated evaluation of treatment progression between sonications should be non-gadolinium^7^.

Diffusion-weighted imaging (DWI) can rapidly detect tissue changes after thermotherapy within minutes^10–12^, offering a potential non-gadolinium method for evaluating MRgFUS treatment between sonications. Research indicates that DWI accurately maps the fibroid ablation area, showing a strong correlation with CE T1-weighted images and histopathological findings^13–15^. However, previous studies did not conduct DWI during treatment or on the treatment-table, and the time elapsed between focused ultrasound treatment and DWI scanning range from a few hours to two days. These limitations hindered the determination of DWI’s value for immediate assessment of the hyperacute changes of thermal ablation. In a previous study utilizing 1.5-T MR, DWI successfully detected necrotic lesions in only 77.8% (35 out of 45) of leiomyomas^16^. As MRgFUS technology has evolved, and it is now mainly guided by 3-T MR, the assessment of DWI via 3-T MR still requires further determination. In this study, we performed DWI on the MRgFUS treatment-table within 15 min post ablation, using 3-T MR, to assess the hyperacute changes in uterine fibroid ablation and to evaluate the potential viability of DWI as an alternative to CE T1-weighted imaging for intra-treatment monitoring.

## Materials and methods

### Patients

This retrospective study was approved by the institutional review board of our Hospital in compliance with ethical principles derived from the Declaration of Helsinki and its subsequent amendments. All participants provided written informed consent. This study analyzed patients who received MRgFUS treatment for uterine fibroids from March 2019 to February 2022. The inclusion criteria included: (1) women aged 18–55 years; (2) patients with symptomatic uterine fibroids confirmed by clinical and MRI, with a maximum diameter of the lesion was ≥ 2.5 cm; (3) patients who underwent MRgFUS treatment; and (4) those who underwent DWI and CE T1-weighted imaging immediately after treatment. Exclusion criteria were: (1) treatment discontinuation; (2) uterine artery embolization, ablation, or surgical treatment of target lesions within 3 months prior to MRgFUS; (3) absence of DWI and/or CE MR examination data; and (4) poor image quality on DWI and/or CE T1-weighted imaging that precluded observation and measurement.

### MRgFUS treatment

Treatment was performed using the MRgFUS system (ExAblate2100, InSightec, Haifa, Israel) , guided by a 3-T MR system (Discovery 750W, GE Healthcare, Milwaukee, USA) equipped with a phased-array body coil for pelvic imaging. Patients were positioned prone on treatment-table, which allowed for MR scans and focused ultrasound surgery. The physician firstly planned the treatment area, which was then subdivided into sonication spots by the computer. The physician targeted the leiomyoma lesion with focused ultrasound, treating each spot individually under MR imaging guidance. After completing all designated sonication spots, the treatment was considered finished.

### MR imaging

Since CE scans are not recommended until after completion of the MRgFUS ablation, our study opted to conduct DWI and CE scans immediately following the final treatment point. The same MR system and body coil employed for MRgFUS were also utilized for the on treatment-table MRI. An axial DWI scan was performed within 15 min post-MRgFUS ablation, followed by CE MR imaging at the identical central position. We utilized the reduced field-of-view (r-FOV) technique for DWI with a b-value of 800 s/mm^2^, based on our prior experience. After administering gadolinium-based contrast agents (0.1 mmol/kg; Gadoteric Acid Meglumine, Hengrui Pharmaceutical, Lianyungang, China) intravenously, we conducted triplanar FSPGR T1-weighted scanning. Table 1 presented the scanning parameters for axial plane CE MR imaging and DWI.

**Table 1:** Magnetic resonance imaging parameters.

Parameters	DWI	CE T1-weighted
Imaging plane	Axial	Axial
Contrast enhanced	No	Yes
Sequence	ss-EPI	FSPGR
TR/TE (ms)	4000.0/72.1	250.0/1.9
b value (s/mm^2^)	800	No
FOV (cm)	36.0 × 18.0	36.0 × 36.0
Frequency direction	R/L	R/L
Section thickness (mm)	5	5
Intersection gap (mm)	1	1
Pixel size (mm^2^)	2.2 × 2.2	1.4 × 2.8
Shim	On	On
Fat suppression	Fat sat	Fat sat
NEX	12	2
Acquisition time	2 min 28 s	1 min 6 s

*DWI* diffusion-weighted imaging, *CE* contrast-enhanced, *ss-EPI* single-shot echo-planar imaging, *FSPGR* fast spoiled gradient recalled, *TR* time of repetition, *TE* time of echo, *FOV* field of view, *NEX* number of excitation.

### Images analysis

The non-enhanced zone of CE T1-weighted imaging was defined as the ablated necrotic lesion created by MRgFUS treament^6,7^. A radiologist with over 20 years of experience in pelvic MR reviewed the post-ablation CE T1-weighted imaging and numbered the leiomyomas. For analysis, axial CE T1-weighted imaging and corresponding DWI images of each leiomyoma were selected.

**Figure 1:**
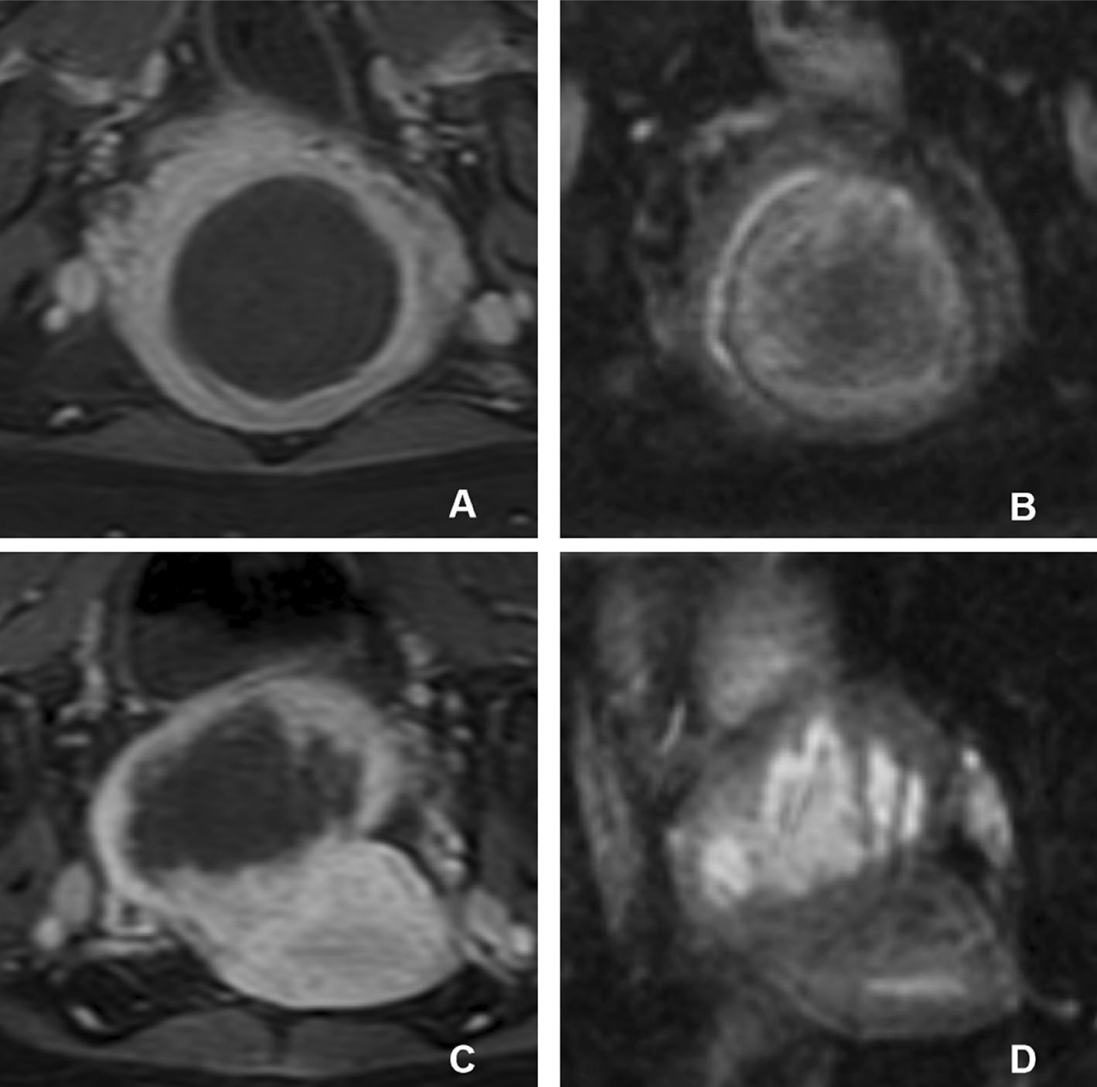
Classification of DWI signal types in ablated necrotic lesions. (**A**,**C**) Contrast-enhanced T1-weighted images post-ablation reveal non-enhancing hypointense zones indicative of ablated necrotic uterine leiomyomas. (**B**,**D**) Corresponding DWI images post-ablation. (**B**) The central hypointense lesion with a surrounding hyperintense ring in the same lesion as in (**A**) is characteristic of type 1, known as the “bull’s eye sign”. (**D**) The inhomogeneous hyperintense lesion lacking a ring in the same lesion as in (**C**) is classified as type 2, referred to as the “bright patch sign”. *DWI* diffusion-weighted imaging.

Two experienced radiologists (observer 1 and observer 2), each with over 20 years of experience in pelvic MRI, independently analyzed the axial CE T1-weighted and DWI images of in a picture archiving and communication system (YLZ Information Technology, Xiamen, China). To minimize image interactions, the radiologists evaluated DWI and CE images separately with a 2 week interval. They were blinded to the patient information during analysis. Prior to interpretation, the researchers discussed another separate set of pre-experimental images, which included 15 fibroids from 10 patients. They reached a consensus on classifying the abnormal signal intensity of ablated necrotic lesions into two types based on DWI features (Fig. 1):

Type 1 ablated necrotic region exhibited a central hypointense lesion with a hyperintense peripheral ring, known as the “bull’s eye sign”, while type 2 appeared as inhomogeneous hyperintense lesions without a ring, known as the “bright patch sign” .

**Figure 2:**
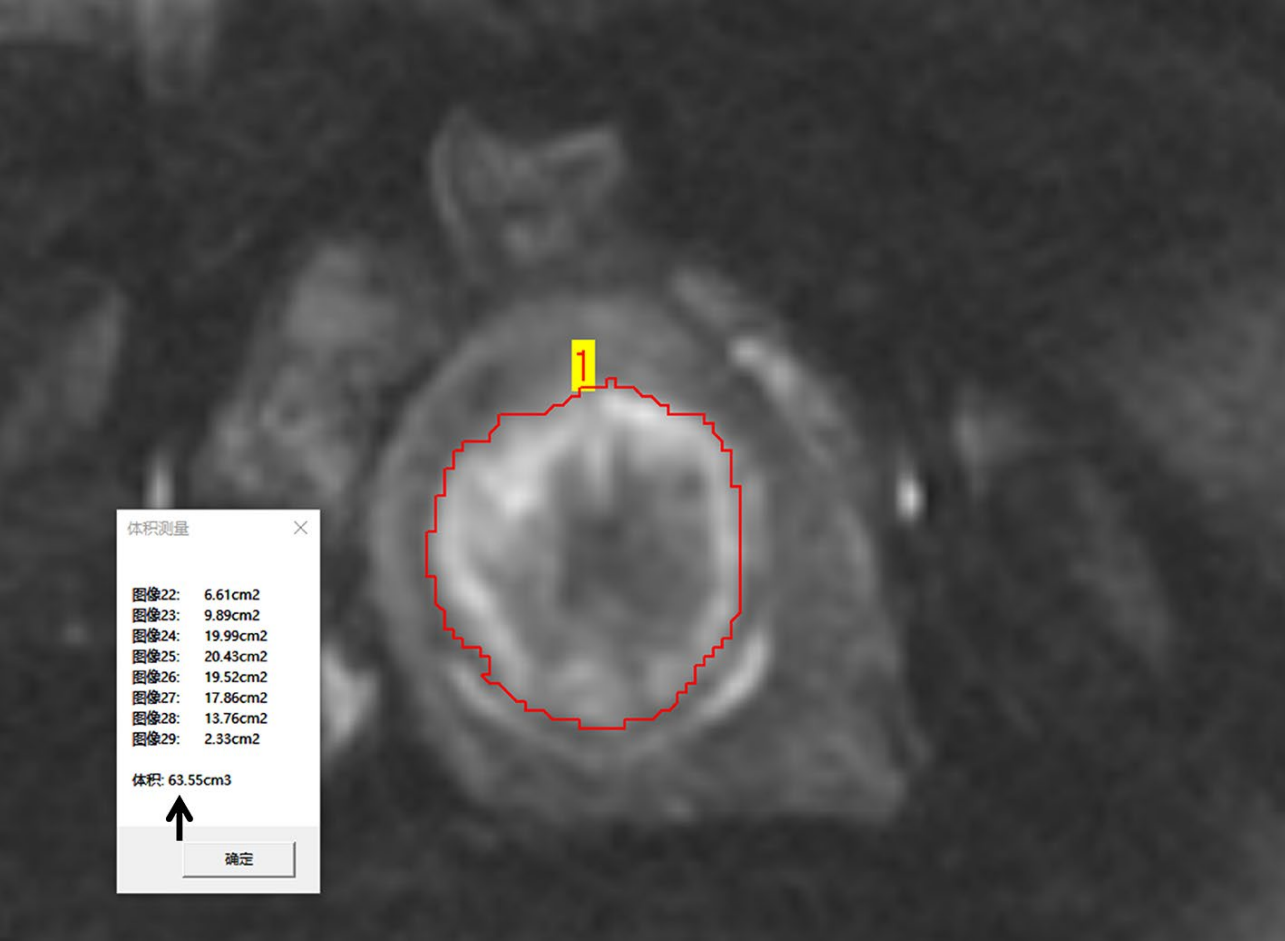
Volume measurement method. The radiologist initially identifies the boundaries of the ablated necrotic area on the images. Then, the area is meticulously outlined layer by layer with precision. Finally, the workstation’s volumetric measurement function is used to automatically calculate the necrotic area’s volume. The illustrated volume of the necrotic region on DWI is 63.55 cm^3^ (arrow). *DWI* diffusion-weighted imaging.

The boundaries of the ablative necrotic area on DWI or CE images were independently defined by observers. They precisely outlined the area layer by layer and calculated the necrotic area’s volume using the workstation’s volumetric measurement function (Fig. 2). In our study, the volume of the ablative necrotic area on DWI or CE images was considered as NPV, and the NPV ratio was calculated as follows: NPV ratio = NPV/fibroid volume.

### Statistical analysis

Continuous variables were presented as mean ± standard deviation (SD). Continuous variables were compared using the two independent-sample or paired *t* test. The intraclass correlation coefficient (ICC) was used to analyze the interobserver consistency of DWI signal types, with consistency was categorized as poor (< 0.50), moderate (0.51–0.75), good (0.76–0.90), or excellent (0.91–1.00). Bland–Altman plot was constructed to provide visual information about the distribution of agreement between DWI and CE measurement, with the 95% limits of agreement (LOA, mean ± 1.96 SD) reported^17^. Statistical analysis was conducted using SPSS statistics software (version 22; IBM Corporation, Armonk, USA). A *p* value of < 0.05 was considered statistically significant.

## Results

### Patients and uterine fibroids

During the inclusion period, 51 women were enrolled. Five were discontinued due to the bowel located at the the uterus’s front. A safe focused ultrasound pathway that physicians ensured MRgFUS ablation safety could not be established. One case had missing information due to a PACS system malfunction, and another case had poor-quality DWI images that hindered reliable observation and measurement. Other 44 women, with a mean age of 38.2 ± 6.9 years (range: 27–50 years), were analyzed. Among these patients, there were 30 with single fibroids, 10 with two fibroids, 1 with three fibroids, and 3 with four fibroids, resulting in a total of 65 uterine fibroids included in the final analysis. The NPV of these 65 fibroids, as demonstrated by CE MR imaging, ranged from 5.64 to 478.43 cm^3^.

### MR findings of ablated necrotic lesions

**Figure 3:**
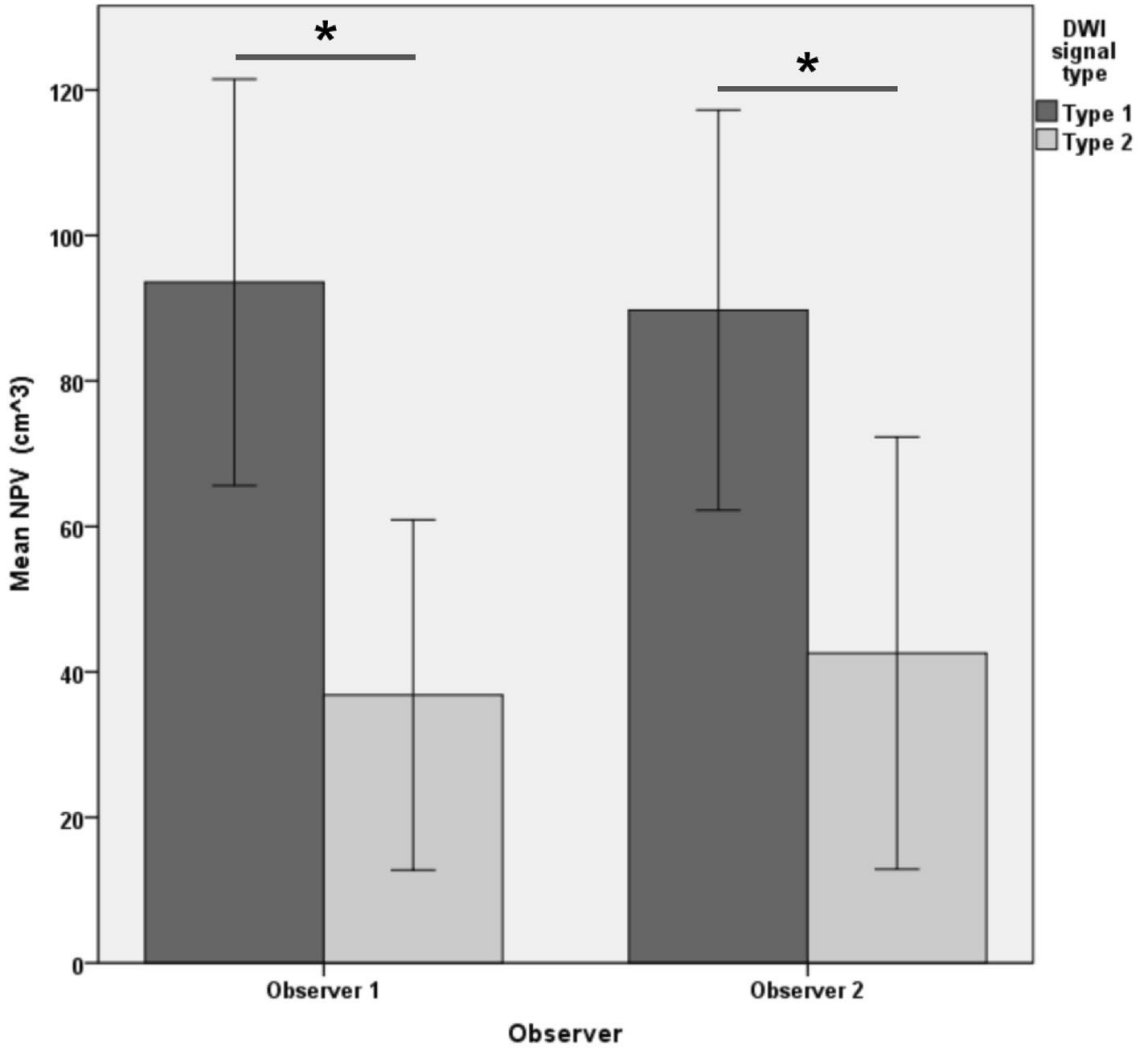
The NPV of different DWI signal type. The NPV of type 1 is larger than that of type 2 lesions. *p < 0.05. *NPV* non-perfused volume, *DWI* diffusion-weighted imaging.

After MRgFUS treatment, ablated lesions of uterine leiomyomas appeared as non-enhancing hypointense zones on CE MR imaging, with surrounding residual leiomyomas showing as enhancing hyperintense zones. The clear contrast between the two zones allowed for easy differentiation. DWI images also displayed abnormal signal areas in each target uterine fibroid, similar in extent to the non-perfused area of CE images. Observers 1 and 2 classified 61.5% (40/65) and 64.6% (42/65) lesions as type 1, respectively. The ICC value for the two observers was 0.804 (95% CI 0.697,0.875; *p* < 0.001). Figure 3 demonstrated the NPV of lesions classified as type 1 and 2, with type 1 having a larger NPV than type 2 lesions (93.56 ± 87.37 cm^3^ vs. 36.83 ± 58.32 cm^3^, *p* = 0.003; and 89.73 ± 88.28 cm^3^ vs. 42.58 ± 68.70 cm^3^, *p* = 0.03).

### Consistency of DWI and CE volumetric measurements

Table 2 and 3 display the volumes of ablated necrotic lesions and the corresponding NPV ratios measured by DWI and CE MR imaging. No significant statistical difference was found between volumes measured by DWI and CE MR imaging or between NPV ratio (all *p* > 0.05). For DWI type 1 leiomyomas, observer 1's NPV ratio measurements using DWI and CE images yielded 92.08% ± 10.90% and 89.33% ± 11.85%, respectively (*p* = 0.051). No significant statistical difference was observed between volumes or other NPV ratio of different DWI signal types (all *p* > 0.05).

**Table 2:** Comparison of volume of ablated necrotic lesions measured by DWI and CE T1-weighted imaging (cm^3^).

Lesions	DWI	CE T1-weighted	*p*-value
All			
Observer 1	73.93 ± 80.93	71.74 ± 81.86	0.102
Observer 2	73.85 ± 81.38	73.05 ± 84.45	0.605
DWI signal type 1			
Observer 1 (n = 40)	96.04 ± 83.76	93.56 ± 87.37	0.225
Observer 2 (n = 42)	89.86 ± 81.57	89.73 ± 88.28	0.951
DWI signal type 2			
Observer 1 (n = 25)	38.56 ± 62.84	36.83 ± 58.32	0.179
Observer 2 (n = 23)	44.61 ± 74.05	42.58 ± 68.70	0.230

*DWI* diffusion weighted imaging, *CE* contrast-enhanced. Values are presented as mean ± SD.

**Table 3:** Comparison of NPV ratio measured by DWI and CE T1-weighted imaging (%).

Lesions	DWI	CE T1-weighted	*p*-value
All			
Observer 1	88.66 ± 15.80	86.41 ± 14.84	0.102
Observer 2	88.09 ± 15.65	86.37 ± 13.96	0.184
DWI signal type 1			
Observer 1 (n = 40)	92.08 ± 10.90	89.33 ± 11.85	0.051
Observer 2 (n = 42)	90.24 ± 13.67	87.89 ± 11.97	0.079
DWI signal type 2			
Observer 1 (n = 25)	83.18 ± 20.53	81.75 ± 17.95	0.613
Observer 2 (n = 23)	84.17 ± 18.44	83.61 ± 16.95	0.841

*DWI* diffusion-weighted imaging, *CE* contrast-enhanced, *NPV* non-perfused volume.

Values are presented as mean ± SD.

**Figure 4:**
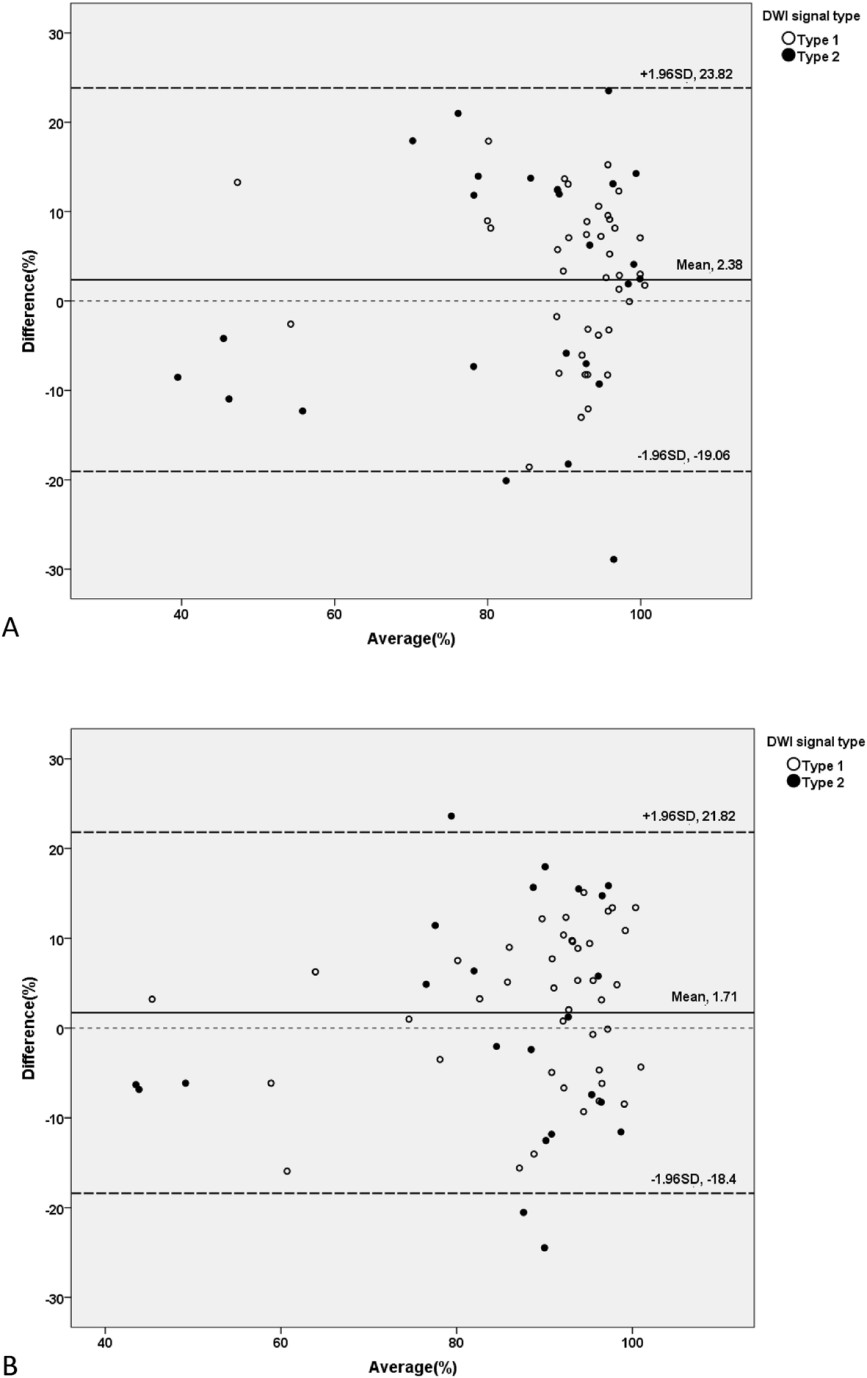
Bland–Altman plots for non-perfused volume ratios. The plots illustrate the difference in non-perfused volume ratios (y-axis) against the mean of the two values (x-axis) as determined by diffusion-weighted imaging (DWI) and contrast-enhanced T1-weighted imaging, with 95% limits of agreement. Measurements were conducted by (**A**) observer 1 and (**B**) observer 2, respectively.

Bland–Altman plots illustrated the difference and average NPV ratio of leiomyomas measured by DWI and CE (Fig. 4). The mean difference and 95% LOA measured by observer 1 were 2.38% and ranged from − 19.06 to 23.82%, respectively. For observer 2, the mean difference was 1.71% and the 95% LOA ranged from − 18.40 to 21.82%. All 5 leiomyomas that exceeding the 95% LOA had type 2 DWI signal.

## Discussion

Accurate and rapid detection of non-perfused zones using non-gadolinium techniques during MRgFUS treatment has long been a necessity and a challenge. In this study, we conducted DWI scans under MRgFUS intraoperative conditions (on treatment-table using 3-T MR) to immediately evaluate the hyperacute changes of thermal ablation in uterine fibroids. The results showed that DWI images provided good lesion visualization, comparable to the non-perfused area of CE MR imaging. This suggests that DWI could serve as a viable non-gadolinium alternative, offering reproducible intra-operative insights that can guide treatment optimization for uterine fibroids.

In this study, we performed the DWI scans using r-FOV and a single b-value, a protocol that presents several advantages for rapid intraoperative monitoring. Unlike the conventional full-FOV DWI technique, r-FOV could improve image quality and reduce artifacts in pelvic DWI^18–21^. During the inclusion period of this study, only one patient was excluded due to poor image quality. In addition, our DWI examination utilized a single b-value of 800 s/mm^2^, which may achieve a better balance between image quality and display capability^18^. Single b-value DWI also offers the benefits of a short scan time (only 2.5 min) and simplicity (no additional complex post-processing required). As a result, it is feasible to perform repeated scans as needed, particularly between sonications. This flexibility is crucial for the treating physician to develop an individualized treatment plans.

Similar to other studies^10–16^, our research demonstrated that DWI images can directly identify ablated necrotic lesions through visual signal changes. In 65 uterine fibroids in the present study, both observers detected all the ablated necrotic lesions confirmed by CE T1-weighted imaging. The abnormal signal intensity of lesions identified by DWI could be categorized into two types: central hypointense lesions with a hyperintense peripheral ring, namely, bull's eye sign or type 1, and inhomogeneous hyperintense lesions without a ring, namely bright patch sign or type 2. The ICC analysis showed good inter-observer consistency for categorizing the type of ablated necrotic lesions on DWI. These two signs provide a straightforward yet reliable DWI characterization that could help physicians accurately and clearly identify ablation necrotic lesions without a CE MR reference.

Histological studies have shown that coagulative necrosis in the target tissues after focused ultrasound thermal ablation, appearing as non-perfused areas on CE MR images^22,23^. Our study found that both hyperintense and hypointense zones on DWI corresponded with non-enhanced areas of ablated necrotic lesions on CE MR images, suggesting that the abnormal signal intensity reflects lesion necrosis. However, a consistent explanation for the complexity of DWI compared to CE MR is still lacking. Our study also showed that DWI signal type correlated with NPV, with type 1 ablation necrotic lesions having a greater NPV than type 2 lesions. This finding may aid in interpreting the complex manifestations on DWI. In MRgFUS treatment, higher total energy always results in a larger NPV. In larger lesions, the central region tends to receive more energy than the periphery due to overlapping sonication spots. It is widely accepted that the DWI signal, using a b value of 800 s/mm^2^, primarily reflects the diffusion of water molecules. Hyperintense areas may indicate cytotoxic edema associated with early ischemia and restricted diffusion of surrounding water molecules^15,24^, while hypointense areas may associated with water liberation following cell membrane rupture caused by intense tissue damage after excessive energy accumulation^13,23,25^. To confirm the accuracy of DWI in detecting necrotic lesions post-ablation, we compared the volume of ablated necrotic lesions and corresponding NPV ratios measured by DWI and CE T1-weighted imaging. Both observers found no significant statistical difference between the volume measured by DWI and CE MR imaging or between NPV ratio. The analysis of DWI signal types 1 and 2 also showed no significant statistical difference between DWI and CE MR imaging volumes, except for type 1 leiomyomas measured by observer 1 (92.08% ± 10.90% vs. 89.33% ± 11.85%, *p* = 0.05). This finding confirmed the excellent agreement between DWI and CE MR imaging in assessing ablated necrotic lesions of uterine fibroids immediately after MRgFUS treatment. In the present study, we used Bland–Altman plots to evaluate the clinical applicability of DWI measurement^17^. The plots indicated that the mean difference of NPV ratios measured by DWI and CE were 2.38% and 1.71%, respectively, with 95% LOA ranging from − 19.06 to 23.82% and − 18.40 to 21.82%. These results suggest that most of the differences were within clinically acceptable limits, indicating that DWI could serve as a potential non-gadolinium alternative to CE MR for evaluating the hyperacute outcome of MRgFUS treatment for uterine fibroids. Combined with the advantages of non-gadolinium, rapid, and simple imaging, DWI may offer a solution to the technical challenges of monitoring treatment progress between sonications. By avoiding the risks associated with gadolinium-based contrast agents, DWI can be performed as often as needed. A reliable intraoperative monitoring technique could have significant clinical implications for optimizing individualized MRgFUS treatment strategies. It can enhance the dynamic and accurate assessment of ablated necrotic lesions, preventing missed ablation area (especially the false positive area) caused by uterine movement and other factors, and improving the NPV ratio.Furthermore, the instantaneous visualization of ablation areas intraoperative can guide the adjustment of treatment targets, preventing unnecessary repetition of ablation in areas already affected by necrosis. This not only enhances treatment efficiency but also mitigates the risks associated with excessive energy accumulation.

As we look to the future, refining DWI accuracy for NPV assessment is a priority. Bland–Altman plots revealed that five leiomyomas outside the 95% LOA had a type 2 DWI signal, indicating a deviation from CE MR. This finding should be considered when analyzing DWI. Future studies should verify whether advanced DWI analysis techniques, such as apparent diffusion coefficient (ADC)^13^, intravoxel incoherent motion^26^, or artificial intelligence^7^, can enhance the accuracy of assessment. Deep learning, in particular, holds promise for enhancing DWI analysis. A study of deep learning-based method for translation of DWI into synthetic CE-T1w scans showed that it allow gadolinium-free visualization of the predicted NPV, potentially allowing for repeated gadolinium-free monitoring of treatment progression during MR-HIFU therapy for uterine fibroids^7^.

The study has several limitations. Firstly, the retrospective design and small sample size may have introduced bias, and ensuring the reliability of blinded analysis was challenging. A prospective, randomized controlled trial is essential to validate our findings. Secondly, due to the focus on intraoperative monitoring, only one b-value was used, and ADC values could not be obtained. Combining multiple b-values and ADC maps is a future research direction. Moreover, the dynamic changes in diffusion effects over time were not captured in our study, as DWI scans were performed only at the end of treatment. Future research should incorporate repeated DWI scans throughout the MRgFUS procedure to monitor these dynamic changes. Finally, the routine use of oxytocin during treatment may affect uterine blood flow, and its impact on DWI performance warrants further investigation.

In conclusion, our study demonstrates that DWI, using a single b-value of 800 s/mm^2^ on 3-T MR system, can effectively delineate ablated necrosis regions based on signal intensity changes. The excellent consistency between DWI and CE MR imaging in the immediate post-treatment assessment of uterine fibroids suggests that DWI can serve as a potential non-gadolinium alternative for evaluating treatment progress between sonications. This has profound implications for personalized MRgFUS treatment strategies, potentially leading to improved clinical outcomes for patients with uterine fibroids.

## Data Availability

The datasets used and/or analyzed during the current study available from the corresponding author on reasonable request.
